# Compression from an enlarged spleen in decompensated cirrhosis mimicking the appearance of fundal varices

**DOI:** 10.1055/a-2106-2135

**Published:** 2023-07-11

**Authors:** Xue-Mei Lin, Chun-Tao Xiao, Xiao-San Hu, Fan Liu, Guo-Dong Yang, Xian-Fei Wang, Cong Yuan

**Affiliations:** 1 Department of Pathology, Institute of Basic Medicine and Forensic Medicine, North Sichuan Medical College, Nanchong, Sichuan, China; 2 Department of Pathology, Affiliated Hospital of North Sichuan Medical College, Nanchong, Sichuan, China; 3 Department of Gastroenterology, Affiliated Hospital of North Sichuan Medical College, Nanchong, Sichuan, China; 4 Department of Gastroenterology, Nanchong Central Hospital, Nanchong, Sichuan, China; 5 Digestive Endoscopy Center, Affiliated Hospital of North Sichuan Medical College, Nanchong, Sichuan, China

Esophagogastric variceal hemorrhage is a medical emergency that requires urgent evaluation and management 1 . Occasionally, submucosal protuberances in the gastric fundus can confuse the prompt recognition of varicose veins 2 . Herein, we report a case of an enlarged spleen compressing the gastric fundus and simulating varicose veins.

A 64-year-old woman with decompensated cirrhosis was admitted to the emergency department with acute hematemesis. Emergency esophagogastroduodenoscopy (EGD), with the patient under conscious sedation, initially indicated esophageal and gastric varices ( Fig. 1 ). Because of poor patient tolerance, endoscopic variceal treatment was not implemented during this procedure, and intravenous vasoactive drug therapy was subsequently commenced. Computed tomography (CT) venography then suggested that the esophageal varices extended below the cardia into the lesser curvature of the stomach, rather being than the fundus ( Fig. 2 ; Video 1 ). Endoscopic ultrasonography (EUS) showed that the subepithelial protrusion in the fundus derived in fact from extraluminal compression ( Fig. 3 ). The CT scan confirmed that extraluminal compression was being caused by the patient’s enlarged spleen ( Fig. 2 a ). Therefore, it became evident that the fundal submucosal protrusion was derived from compression by the enlarged spleen, rather than varicose veins, and the patient was diagnosed with gastroesophageal varices type 1 3 . Esophageal variceal ligation was eventually performed.

**Fig. 1:**
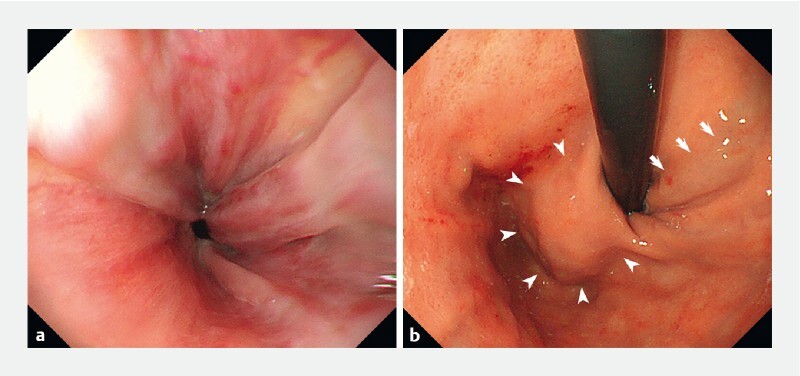
Endoscopic views from an emergency esophagogastroduodenoscopy showing an appearance suggestive of varicose veins in the: **a** esophagus; **b** fundus (arrowheads) and lesser curvature of the gastric body (arrows).

**Fig. 2:**
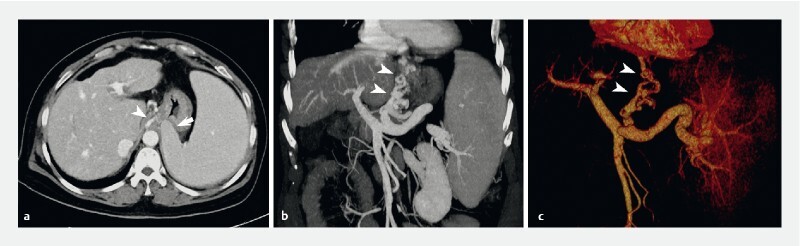
Computed tomography venography of the portal vein system showing the esophageal varices extending below the cardia into the lesser curvature of the stomach (arrowheads), rather than the fundus, with the enlarged spleen seen to be compressing the gastric fundus in image **a** (arrow).

**Video 1** Initial esophagogastroduodenoscopy appears to show esophageal and gastric varices. Computed tomography (CT) venography reveals that the esophageal varices extend below the cardia into the lesser curvature of the stomach, rather than the fundus. Endoscopic ultrasonography shows that the subepithelial protrusion in the fundus is derived from extraluminal compression, and the CT image confirms that this extraluminal compression is caused by the enlarged spleen.


**Fig. 3:**
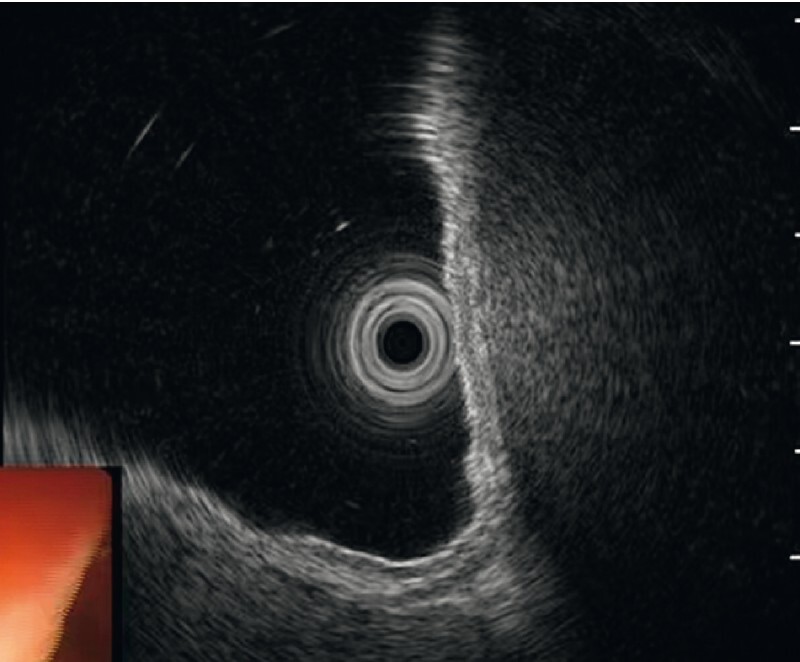
Endoscopic ultrasonography of the gastric fundal protrusion showing that the subepithelial protrusion in the fundus is derived from extraluminal compression.

In patients with liver cirrhosis, not all gastric varices have a serpiginous shape or a venous hue, which poses a challenge in distinguishing submucosal protrusions between gastric varices, submucosal tumors, and thickened mucosal folds on endoscopic visualization alone 2 . Our experience with this case highlights that fundal protrusion can also be caused by external compression from an enlarged spleen in patients with portal hypertension. In this scenario, CT scanning and EUS provide important clues in the accurate identification of gastric fundal protrusions. During emergency EGD, a preliminary differential diagnosis can be approached by perceiving the texture of the protrusion, namely whether there is soft or hard feedback.

Endoscopy_UCTN_Code_CCL_1AB_2AC_3AG

## Notes

Endoscopy E-Videos

https://eref.thieme.de/e-videos
*E-Videos* is an open access online section of the journal *Endoscopy* , reporting on interesting cases and new techniques in gastroenterological endoscopy. All papers include a high-quality video and are published with a Creative Commons CC-BY license. Endoscopy E-Videos qualify for HINARI discounts and waivers and eligibility is automatically checked during the submission process. We grant 100% waivers to articles whose corresponding authors are based in Group A countries and 50% waivers to those who are based in Group B countries as classified by Research4Life (see: https://www.research4life.org/access/eligibility/ ). This section has its own submission website at https://mc.manuscriptcentral.com/e-videos
